# 

**Published:** 1887-04

**Authors:** 


THE AMERICAN MEDICAL ASSOCIATION is appointed to assemble in Chicago June 7th next.

---

SEE advertisement of the great work entitled "Medical and Surgical Memoirs," by Prof. Joseph Jones, of New Orleans.

